# Are Female Patients More Overlooked in the Diagnosis of Fabry Disease?

**DOI:** 10.4274/balkanmedj.galenos.2019.2019.11.148

**Published:** 2020-02-28

**Authors:** Muhammet Gürdoğan, Ömer Ferudun Akkuş

**Affiliations:** 1 Department of Cardiology, Trakya University School of Medicine, Edirne, Turkey

To the Editor,

We have read the article by Barman et al. (1) titled, “The Prevalence of Fabry disease Among Turkish Patients with non-obstructive Hypertrophic Cardiomyopathy: Insights from a Screening Study” with interest. In their descriptive study, the researchers found that the frequency of Fabry disease was 2.5% by screening 80 patients (53 males) diagnosed with hypertrophic cardiomyopathy without left ventricular outflow tract obstruction. The au-thors emphasized that Fabry disease should be kept in mind in the differential diagnosis of patients with unexplained left ventricular hypertrophy (1).

Fabry disease is an X-linked recessive lysosomal storage disorder, first described in 1898, characterized by a deficiency of α-galactosidase A resulting from a genetic mutation. In Fabry disease, glycosphingolipid accumulation occurs in many tissues, especially in the nervous system, heart, kidneys, and skin (2,3). As stated by Barman et al. (1), different results were found on the scans performed in different parts of the world to investigate the incidence of Fabry disease. The actual incidence of Fabry disease is not exactly known, but it is estimated to be much higher than that seen in daily cardi-ology practice. It is known that males are affected more frequently and more seriously because of the X-linked inheritance in Fabry disease. However, it has been reported that heterozygous women are also affected by the disease, but the disease symptoms such as left ventricular hypertrophy appear later in life (about 10 years later) (3,4). Myocardial fibrosis, which is a guide in determining the prognosis of Fabry disease and the patients who will benefit from enzyme replacement therapy, has been reported in women without left ventricular hypertrophy, unlike men (3,5). It is reported in the literature that the variant of Fabry disease characterized only by cardiac involvement is more common in women. Fabry disease screening based solely on left ventricular hypertrophy means that the disease is detected less frequently in females. The fact that left ventricular hypertrophy has been reported in only one out of every three women diagnosed with Fabry disease in the literature supports this idea. (3,4,5).

The study by Barman et al. (1) is valuable for being the first study to investigate the incidence of Fabry disease in patients with unexplained left ventricular hypertrophy in Turkey. However, the under-representation of the female gender in the study group (two out of three patients are males) may cause the frequency of Fabry disease to be both detected and reported lower than it actually is. In the future, large-scale studies that minimize the effect of gender difference will help to deter-mine the true prevalence of Fabry disease.
